# The prevalence and prognostic impact of tumor-infiltrating lymphocytes in uterine carcinosarcoma

**DOI:** 10.1186/s12885-021-09026-6

**Published:** 2021-12-07

**Authors:** Jesse Lopes da Silva, Lucas Zanetti de Albuquerque, Fabiana Resende Rodrigues, Guilherme Gomes de Mesquita, Cláudia Bessa Pereira Chaves, Martín Hernán Bonamino, Andreia Cristina de Melo

**Affiliations:** 1 Division of Clinical Research and Technological Development, Brazilian National Cancer Institute, Rio de Janeiro, Brazil; 2 Gynecologic Oncology Section, Brazilian National Cancer Institute, Rio de Janeiro, Brazil; 3 Division of Pathology, Brazilian National Cancer Institute, Rio de Janeiro, Brazil; 4 Immunology and Tumor Biology Program, Brazilian National Cancer Institute, Rio de Janeiro, Brazil; 5 Vice-Presidency of Research and Biological Collections (VPPCB), Oswaldo Cruz Foundation (FIOCRUZ), Rio de Janeiro, Brazil

**Keywords:** Uterine carcinosarcoma, Tumor-infiltrating lymphocyte, Tumor microenvironment, Immune biomarkers, Tumor microenvironment

## Abstract

**Objective:**

To examine the prevalence and prognostic role of tumor microenvironment (TME) markers in uterine carcinosarcoma (UCS) through immunohistochemical characterization.

**Methods:**

The internal database of our institution was queried out for women with UCS who underwent surgery and thereafter postoperative chemotherapy with carboplatin and paclitaxel between January 2012 and December 2017. Tissue microarrays containing surgical samples of UCS from 57 women were assessed by immunohistochemistry for CD3, CD4, CD8, FOXP3, PD-1, PD-L1, and PD-L2.

**Results:**

The mean age was 65.3 years (range, 49 to 79 years). For the epithelial component (E), CD3_E and CD4_E were highly expressed in 38 (66.7%) and in 40 (70.1%) patients, respectively, and were significantly associated with more advanced stages (*p* = 0.038 and *p* = 0.025, respectively). CD8_E was highly expressed in 42 (73.7%) patients, FOXP3_E 16 (28.1%), PD-1_E 35 (61.4%), PD-L1_E 27 (47.4%) and PD-L2_E 39 (68.4%). For the sarcomatous component (S), the prevalence of high expression was: CD3_S 6 (10.5%), CD4_S 20 (35.1%), CD8_S 44 (77.2%), FOXP3_S 8 (14%), PD-1_S 14 (24.6%), PD-L1_S 14 (24.6%) and PD-L2_S 8 (14%). By multivariate analysis, the CD8/FOXP3_S ratio (*p* = 0.026), CD4_E (*p* = 0.010), PD-L1_E (*p* = 0.013) and PD-L1_S (*p* = 0.008) markers significantly influenced progression-free survival. CD4/FOXP3_S ratio (*p* = 0.043), PD-1_E (*p* = 0.011), PD-L1_E (*p* = 0.036) and PD-L1_S (*p* = 0.028) had a significant association with overall survival.

**Conclusion:**

Some differences in UCS clinical outcomes may be due to the subtype of TILs and PD-1/PD-L1 axis immune checkpoint signaling.

**Supplementary Information:**

The online version contains supplementary material available at 10.1186/s12885-021-09026-6.

## Introduction

Uterine carcinosarcomas (UCS) are uncommon and overly aggressive tumors with biphasic histology composed of epithelial (E) and sarcomatous (S) elements [1, 2]. Recently, these tumors have been thought to be derived from monoclonal carcinoma cells branched from embryonal mesoderm [3]. Given that, UCS are pointed out as a model for epithelial-mesenchymal transition, a mechanism that results in loss of cell polarity, adhesion, migratory and invasive properties, which facilitates metastasis [4].

UCS account for less than 5% of uterine cancers and the incidence ranges from 5.1 to 6.9 per 1,000,000 person-years worldwide [5]. This tumor is usually diagnosed in older women, with a median age ranging from 62 to 67 years [6]. African-American women are significantly at more risk of having UCS compared to Caucasian women [7]. In contrast, there is no Brazilian data regarding UCS specifically.

The tumor microenvironment (TME) plays an important role in the progression and metastasis of tumors through the so-called “cancer immunoediting” mechanism that leads to escape of cancer cells from immune surveillance [8]. Several cell types of the innate and adaptive immune system are involved in this complex process, including CD8+ lymphocytes, Th1/Th2 subclasses of CD4+ T lymphocytes, natural killer (NK) cells and forkhead box protein 3 (FOXP3+) T regulatory (Treg) cells [9]. Recently, much has been discussed about the subtyping of tumor-infiltrating lymphocytes (TILs) in neoplasms of different sites [10].

The programmed death 1 receptor (PD-1), found on the surface of activated T cells and many other immune cells, is currently one of the most studied immune regulatory pathways able to strongly influence the mechanism of carcinogenesis, with a great potential of prognostic and therapeutic effect [11]. PD-1 with its ligands, programmed death-ligand 1 (PD-L1) and programmed death-ligand 2 (PD-L2), play a crucial role in tumor immune evasion. The immune checkpoint pathways inhibit T cell receptor (TCR) signaling via engagement of SHP-1 and SHP-2 phosphatases, resulting in reduced T-cell proliferation and cytokine production, increasing susceptibility to apoptosis [12]. The Inhibition of the PD-1/PD-L1/PD-L2 interaction by different immunological therapies can cause the T cell function to be restored, providing enhanced anti-tumor immune responses [13].

So far, no effective immune biomarkers have been timely assessed for UCS. This cohort aimed to gain a better insight into the prevalence and prognostic value of TILs subtype, and also PD-1, PD-L1 and PD-L2 expression in patients with UCS.

## Materials and methods

### Patient selection and data collection

This study was approved by the Ethics in Human Research Committee of the Brazilian National Cancer Institute (INCA), Rio de Janeiro, Brazil, and was conducted following the Good Clinical Practice Guidelines. All women diagnosed with UCS, who underwent surgery and thereafter postoperative chemotherapy with the standard dose of every-3-week carboplatin AUC 5 and paclitaxel 175 mg/m^2^ (CP) for six cycles [14] at INCA between January 2012 and December 2017, were identified through the internal database. Patients with a scarce or inadequate pathological sample, with synchronous or anachronistic tumors, were excluded from this cohort. Clinical data regarding sociodemographic factors, staging, surgery, histological subtype (homologous versus heterologous), progression and survival were retrospectively obtained in the medical records. The staging was performed based on the criteria of the International Federation of Gynecology and Obstetrics (FIGO, 2009) [15].

### Immunohistochemistry

The tissue microarray (TMA) was built using samples of stromal areas of greatest tumor cellularity present in formalin-fixed paraffin-embedded primary tumors in surgical specimens. Three cores were punched in each of the two tumor components (E and S). All immunohistochemistry (IHC) analyses were performed on 4-μm sections following standard procedures. TMA samples were immunostained for CD3 (clone MRQ-39, Cell Marque, diluted 1:1000), CD4 (clone SP35, Cell Marque, diluted 1:400), CD8 (clone SP 16, Cell Marque, diluted 1:1000), FOXP3 (clone 236A/E7, Abcam, diluted 1:50), PD-1 (clone NAT105, Cell Marque, diluted 1:100), PD-L1 (clone SP142, Ventana, prediluted) and PD-L2 (clone ab200377, Abcam, diluted 1:200). The tumor cell staining was compared with that of negative controls made from counterstaining with hematoxylin and positive controls.

Intratumoral stromal immune markers were manually counted and scored as described hereafter. For PD-L1, PD-L2 and PD-1, the slides were scored according to the percentage of positive immune and tumor cells divided by the number of fields to calculate the mean value for each case, determined at 40x magnification [16]. For TILs subpopulations (CD3+, CD4+, CD8+ and FOXP3+) intratumoral stromal lymphocytes were counted manually and quantified as the average absolute number of immunolabeled lymphocytes at each observed field at 40x magnification [17].

For statistical purposes, the scores of these biomarkers were dichotomized into low and high-level groups for each of the histological elements, E and S, based on cut-off points calculated according to the surv_cutpoint function of the survminer R package [18]. Thus, the cut-off for CD 3_E was 0, CD3_S was 60, CD4_E was 0, CD4_S was 20, CD8_E was 0, CD8_S was 1, FOXP3_E was 0, FOXP3_S was 5, PD-1_E was 0, PD-1_S was 1, PD-L1_E was 1, PD-L1_S was 20, PD-L2_E was 40 and PD-L2_S was 90. Likewise, the cutoff for ratios: CD4/FOXP3_E+ was 1, CD4/FOXP3_S+ was 2, CD8/FOXP3_E+ was 3, CD8/FOXP3_S+ was 3.7, CD8/CD4_E+ was 0.18 and CD8/CD4_S+ was 3. The boxplots shown in additional Fig. 1 represent the distributions of the values of markers evaluated. The pathological analysis was performed twice for each slide of TMA by two experienced pathologists.

### Statistical analysis

Progression-free survival (PFS) was calculated from the date of first CP infusion to the earliest date of disease progression, recurrence, or death. Overall survival (OS) was calculated from the first CP infusion to the date of death of any cause or censored if the patient was known to be alive on the last day of data collection. The Kaplan-Meier method was used to estimate PFS and OS for each variable. Patients were stratified by age, body mass index (BMI), race, stage, omentectomy, residual disease, adjuvant radiotherapy, lymphovascular invasion (LVI), histological subtype and IHC markers status. All continuous variables were evaluated by the Shapiro-Wilk test of normality. Categorical variables were described by their absolute and relative frequencies.

To assess the association of the IHC markers scores with mean age and BMI, the Student’s t-test was used. The correlation with categorical clinicopathological parameters was performed by Pearson’s chi-squared test and, when applicable, by Fisher’s exact test. A further analysis comparing the paired scores of immunohistochemistry factors for epithelial and sarcomatous components was performed by the Wilcoxon signed-rank test. The crude Hazard Ratio (HR) for each variable was calculated by the Cox proportional hazards. The variables evaluated for survival outcomes on univariate analysis were adjusted for the FIGO stage in multivariate models. A *p*-value < 0.05 was considered statistically significant. The missing data were excluded from the analysis. The statistical analyses were conducted using the R project version 3.5.3 [18].

## Results

The clinicopathological and IHC data of the 57 women included in this cohort were summarized in Tables 1 and 2. The mean age was 65.3 years (range, 49 to 79 years). Briefly, there was a predominance of women ≥60 years old (40 cases, 70.2%), non-Caucasians (42 cases, 75%), with advanced disease (42 cases, 73.7%), heterologous subtype (30 cases, 71.4%), and LVI was detected in 25 cases (60.1%) (additional Table 1). As for treatment data, 37 (65%) patients were submitted to lymphadenectomy, 27 (47.4%) patients underwent omentectomy, optimal debulking (defined as residual disease < 1.0 cm) was achieved in 39 (68.4%) and adjuvant radiotherapy was provided to 24 (42.8%) patients (additional Table 2).

**Table 1:** Clinicopathological characteristics of the epithelial component of carcinosarcoma and the status of intratumoral stromal CD3, CD4, CD8, FOXP3, PD-1, PD-L1 and PD-L2 (*N* = 57)

Clinicopathological features	Total no. of cases	CD3_E			CD4_E			CD8_E			FOXP3_E			PD-1_E			PD-L1_E			PD-L2_E		
		Low	High	p-value	Low	High	*p*-value	Low	High	p-value	Low	High	p-value	Low	High	p-value	Low	High	p-value	Low	High	p-value
Age *N* = 57																						
< 60	17	6	11	0.838	2	15	0.052	3	14	0.513	11	6	0.523	7	10	0.138	**5**	**12**	**0.022**	1	16	0.413
≥ 60	40	13	27		15	25		12	28		30	10		25	15		**25**	**15**		7	33	
Stage *N* = 57																						
I/II	15	**2**	**13**	**0.038**	**2**	**13**	**0.025**	5	10	0.584	10	5	0.697	6	9	0.138	8	7	0.587	**0**	**15**	**0.041**
III/IV	42	**17**	**25**		**15**	**27**		10	32		31	11		26	16		22	20		**8**	**34**	
Histological subtype *N* = 42																						
Heterologous	30	9	21	0.292	12	18	0.277	8	22	0.715	23	7	0.699	18	12	0.740	21	9	0.158	5	25	0.298
Homologous	12	6	6		2	10		4	8		8	4		8	4		5	7		0	12	
LVI *N* = 41																						
Present	25	4	12	1.000	3	13	0.712	7	9	0.053	12	4	0.734	13	12	0.790	13	12	0.606	4	21	1.000
Absent	16	6	19		7	18		3	22		17	8		9	7		7	9		2	14	

Differences in absolute value correspond to missing data. Significant *P*-values are emboldened

*LVI* Lymphovascular Invasion, *E* Epithelial Component, *ER* Estrogen Receptor, *PR* Progesterone Receptor, *CD3* Cluster of Differentiation 3, *CD4* Cluster of Differentiation 4, *CD8* Cluster of Differentiation 8, *FOXP3* Forkhead Box P3, *PD-1* Programmed Cell Death Protein 1, *PD-L1* Programmed Death-Ligand 1, *PD-L2* Programmed Death-Ligand 2

**Table 2:** Clinicopathological characteristics of the sarcomatous component of carcinosarcoma and the status of intratumoral stromal CD3, CD4, CD8, FOXP3, PD-1, PD-L1 and PD-L2 (*N* = 57)

Clinicopathological features	Total no. of cases	CD3_S			CD4_S			CD8_S			FOXP3_S			PD-1_S			PD-L1_S			PD-L2_S		
		Low	High	*p*-value	Low	High	*p*-value	Low	High	*p*-value	Low	High	*p*-value	Low	High	*p*-value	Low	High	*p*-value	Low	High	*p*-value
Age *N* = 57																						
< 60	17	14	3	0.503	8	9	0.124	3	14	0.795	14	3	0.924	12	5	0.827	10	7	0.118	14	3	0.924
≥ 60	40	37	3		19	11		10	30		35	5		31	9		33	7		35	5	
Stage *N* = 57																						
I/II	15	12	3	0.367	11	4	0.631	5	10	0.439	12	3	0.732	10	5	0.569	10	5	0.569	13	2	1.000
III/IV	42	39	3		26	16		8	34		37	5		33	9		33	9		36	6	
Histological subtype *N* = 42																						
Heterologous	30	28	2	0.909	22	8	0.957	7	23	0.418	25	5	0.834	24	6	0.647	25	5	1.000	27	3	0.940
Homologous	12	12	0		8	4		5	7		11	1		11	1		10	2		10	2	
LVI *N* = 41																						
Present	25	21	4	0.252	14	11	0.625	3	22	0.266	20	5	0.844	17	8	0.567	18	7	0.434	21	4	0.760
Absent	16	16	0		11	5		5	11		14	2		13	3		14	2		12	4	

Differences in absolute value correspond to missing data. Significant *P*-values are emboldened

*LVI* Lymphovascular Invasion, *S* Sarcomatous Component, *ER* Estrogen Receptor, *PR* Progesterone Receptor, *CD3* Cluster of Differentiation 3, *CD4* Cluster of Differentiation 4, *CD8* Cluster of Differentiation 8, *FOXP3* Forkhead Box P3, *PD-1* Programmed Cell Death Protein 1, *PD-L1* Programmed Death-Ligand 1, *PD-L2* Programmed Death-Ligand 2

By analyzing the E component, CD3_E was highly expressed in 38 (66.7%) patients and significantly associated with more advanced stages (*p* = 0.038). CD4_E was highly expressed in 40 (70.1%) patients and was significantly associated with more advanced stages (*p* = 0.025). CD8_E, FOXP3_E and PD-1_E were at a high level in 42 (73.7%), 16 (28.1%) and 25 (43.9%) patients, respectively, but did not show significant association with any of the clinicopathological features. PD-L1_E was overexpressed in 27 (47.4%) patients and was significantly more highly expressed in patients ≥60 years old (*p* = 0.022) (Table 1). As for the assessment of IHC markers in the S component, the frequencies of highly positive expression were much lower in CD3_S (6 cases, 10.5%) and CD4_S (20 cases, 35.1%). CD8_S was expressed in 44 cases (77.2%), FOXP3_S in 8 cases (14%), PD-1_S in 14 cases (24.6%), PD-L1_S in 14 cases (24.6%) and PD-L2_S in 8 cases (14%) (Table 2). Except for PD-L1, all other TME markers (CD3, CD4, CD8, FOXP3, PD-1 and PD-L2) showed significantly greater expression in the sarcomatous component than in the epithelial component (additional Table 5). Additional Fig. 2 shows representative images of cases with high expression of IHC markers.

With a median follow-up of 51 months (95% confidence interval, CI: 40–70), 42 patients had disease progression or died until the moment of the analysis, and the three-year rate of progression-free survival in the general study population was 21.2% (95% CI: 11.7–38.1). The outcome PFS was compared according to the clinicopathological parameters and IHC evaluations. As stated by the data in Table 3, patients with early stages I/II had 63% lower risk of progression than advanced stages III/IV (Hazard ratio, HR 0.37; 95% CI: 0.16–0.84; *p* = 0.017). By multivariate analysis for PFS, patients with high expression of CD4_E (high vs low; HR 0.43; 95% CI: 0.23–0.82; *p* = 0.010), PD-L1_E (high versus low; HR 0.45; 95% CI: 0.24–0.84; *p* = 0.013) and PD-L1_S (high versus low; HR 0.30; 95% CI: 0.12–0.74; *p* = 0.008) had significantly lower risk of progression or death. Conversely, patients with residual disease after surgery (R1/2 versus R0; HR 3.09; 95% CI: 1.34–7.08; *p* = 0.008) and high CD8/FOXP3_S ratio (high versus low; HR 2.05; 95% CI: 1.08–3.85; *p* = 0.026) significantly yielded poorer OS.

**Table 3:** Crude and adjusted Hazards Ratios for Carcinosarcoma progression-free survival (PFS) estimated by univariate analysis and multivariate analysis

Clinicopathological features	Univariate analysis			Multivariate analysis		
	HR	95%CI	*p*-value	HR	95%CI	*p*-value
Age (< 60 vs. ≥ 60)	1.03	0.99–1.07	0.171	1.03	0.99–1.07	0.111
**Stage (I/II vs III/IV)**	**0.37**	**0.16–0.84**	**0.017**	–	–	–
**Residual disease (R1/2 vs R0)**	**4.19**	**2.13–8.25**	**0.001**	**3.09**	**1.34–7.08**	**0.008**
Adjuvant radiotherapy (Yes vs No)	0.57	0.30–1.08	0.087	0.66	0.34–1.26	0.213
LVI (present vs absent)	1.06	0.50–2.25	0.872	1.05	0.49–2.22	0.906
Histological subtype (Homologous **vs** Heterologous)	0.83	0.39–1.78	0.631	0.94	0.44–2.02	0.885
CD3_E (high vs low)	0.58	0.31–1.08	0.086	0.67	0.26–1.27	0.229
CD3_S (high vs low)	0.48	0.15–1.57	0.225	0.62	0.19–2.07	0.439
**CD4_E (high vs low)**	**0.39**	**0.21–0.74**	**0.004**	**0.43**	**0.23–0.82**	**0.010**
CD4_S (high vs low)	0.62	0.80–3.21	0.184	0.60	0.30–1.20	0.153
CD8_E (high vs low)	0.67	0.35–1.29	0.232	0.61	0.31–1.19	0.151
CD8_S (high vs low)	0.86	0.42–1.75	0.669	0.71	0.34–1.48	0.364
FOXP3_E (high vs low)	0.55	0.26–1.17	0.120	0.54	0.26–1.16	0.115
FOXP3_S (high vs low)	0.41	0.14–1.15	0.090	0.42	0.15–1.19	0.103
**PD-1_E (high vs low)**	**0.47**	**0.25–0.90**	**0.022**	0.54	0.28–1.04	0.065
PD-1_S (high vs low)	0.66	0.31–1.41	0.286	0.73	0.34–1.56	0.420
**PD-L1_E (high vs low)**	**0.47**	**0.25–0.88**	**0.019**	**0.45**	**0.24–0.84**	**0.013**
**PD-L1_S (high vs low)**	**0.28**	**0.12–0.67**	**0.004**	**0.30**	**0.12–0.74**	**0.008**
PD-L2_E (high vs low)	0.48	0.22–1.06	0.070	0.62	0.28–1.38	0.241
PD-L2_S (high vs low)	0.46	0.16–1.31	0.147	0.43	0.15–1.22	0.114
CD4/FOXP3_E ratio (high vs low)	0.57	0.31–1.04	0.067	0.63	0.34–1.16	0.141
CD4/FOXP3_S ratio (high vs low)	1.43	0.76–2.67	0.267	1.65	0.87–3.11	0.120
CD8/FOXP3_E ratio (high vs low)	0.74	0.39–1.41	0.363	0.84	0.44–1.60	0.605
**CD8/FOXP3_S ratio (high vs low)**	**2.27**	**1.21–4.26**	**0.010**	**2.05**	**1.08–3.85**	**0.026**
CD8/CD4_E ratio (high vs low)	0.61	0.24–1.56	0.299	0.51	0.20–1.33	0.171
CD8/CD4_S ratio (high vs low)	1.69	0.78–3.67	0.186	1.87	0.86–4.10	0.113

All variables were adjusted for staging in multivariate analysis. Significant *P*-values are emboldened

*LVI* Lymphovascular Invasion, *E* Epithelial Component, *S* Sarcomatous Component, *ER* Estrogen Receptor, *PR* Progesterone Receptor, *CD3* Cluster of Differentiation 3, *CD4* Cluster of Differentiation 4, *CD8* Cluster of Differentiation 8, *FOXP3* Forkhead Box P3, *PD-1* Programmed Cell Death Protein 1, *PD-L1* Programmed Death-Ligand 1, *PD-L2* Programmed Death-Ligand 2

By the moment of the analysis, 38 patients died, and the three-year OS rate was 29.4% (95% CI: 18.1–47.6). As shown in Table 4, patients with early-stage disease I/II had a risk of death 72% lower than advanced stages III/IV (HR 0.28; 95% CI: 0.11–0.71; *p* = 0.008). Regarding multivariate analysis for OS, patients with high expression of PD1_E (high vs low; HR 0.39; 95% CI: 0.19–0.81; *p* = 0.011), PD-L1_E (high versus low; HR 0.49; 95% CI: 0.25–0.96; *p* = 0.037) and PD-L1_S (high versus low; HR 0.37; 95% CI: 0.15–0.90; *p* = 0.028) had significantly lower risk of death. By contrast, there was significantly worse prognosis for patients with incomplete debulking (R1/2 versus R0; HR 2.87; 95% CI: 1.40–5.89; *p* = 0.003) and low CD4/FOXP3_S ratio (high versus low; HR 2.04; 95% CI: 1.02–4.09; *p* = 0.043).

**Table 4:** Crude and adjusted Hazards Ratios for Carcinosarcoma Overall survival (OS) estimated by univariate analysis and multivariate analysis

Clinicopathological features	Univariate analysis			Multivariate analysis		
	HR	95%CI	*p*-value	HR	95%CI	*p*-value
Age	1.02	0.98–1.07	0.307	1.03	0.98–1.08	0.157
**Stage (I/II vs III/IV)**	**0.28**	**0.11–0.71**	**0.008**	–		
**Residual disease (R1/2 vs R0)**	**3.83**	**1.92–7.62**	**0.001**	**2.87**	**1.40–5.89**	**0.003**
Adjuvant radiotherapy (Yes vs No)	0.53	0.27–1.05	0.068	0.64	0.32–1.28	0.214
LVI (present vs absent)	1.05	0.47–2.35	0.900	0.99	0.44–2.22	0.985
Histological subtype (Homologous vs Heterologous)	0.84	0.38–1.87	0.671	0.90	0.40–2.01	0.806
**CD3_E (high vs low)**	**0.52**	**0.27–0.99**	**0.047**	0.65	0.33–1.25	0.200
CD3_S (high vs low)	0.54	0.16–1.79	0.315	0.73	0.22–2.42	0.605
CD4_E (high vs low)	0.52	0.26–1.01	0.054	0.56	0.28–1.11	0.972
CD4_S (high vs low)	0.66	0.32–1.33	0.241	0.60	0.30–1.22	0.160
CD8_E (high vs low)	0.69	0.35–1.39	0.301	0.60	0.29–1.23	0.163
CD8_S (high vs low)	0.77	0.37–1.59	0.478	0.61	0.29–1.28	0.193
FOXP3_E (high vs low)	0.46	0.20–1.06	0.069	0.43	0.18–1.01	0.052
**FOXP3_S (high vs low)**	**0.24**	**0.06–0.99**	**0.048**	0.26	0.06–1.09	0.066
**PD-1_E (high vs low)**	**0.33**	**0.16–0.68**	**0.002**	**0.39**	**0.19–0.81**	**0.011**
PD-1_S (high vs low)	0.46	0.19–1.10	0.080	0.52	0.22–1.27	0.152
**PD-L1_E (high vs low)**	0.54	0.28–1.03	0.062	**0.49**	**0.25–0.96**	**0.037**
**PD-L1_S (high vs low)**	**0.34**	**0.14–0.81**	**0.015**	**0.37**	**0.15–0.90**	**0.028**
PD-L2_E (high vs low)	0.56	0.24–1.29	0.173	0.76	0.32–1.77	0.519
PD-L2_S (high vs low)	0.44	0.13–1.44	0.174	0.41	0.12–1.35	0.144
CD4/FOXP3_E ratio (high vs low)	0.67	0.36–1.27	0.223	0.76	0.40–1.45	0.407
**CD4/FOXP3_S ratio (high vs low)**	1.83	0.92–3.65	0.086	**2.04**	**1.02–4.09**	**0.043**
CD8/FOXP3_E ratio (high vs low)	0.68	0.35–1.33	0.262	0.79	0.40–1.57	0.506
**CD8/FOXP3_S ratio (high vs low)**	**2.22**	**1.14–4.29**	**0.018**	1.91	0.98–3.73	0.058
CD8/CD4_E ratio (high vs low)	0.69	0.27–1.78	0.446	0.58	0.23–1.51	0.268
CD8/CD4_S ratio (high vs low)	0.98	0.39–2.53	0.969	1.16	0.45–3.01	0.754

All variables were adjusted for staging in multivariate analysis. Significant *P*-values are emboldened

*LVI* Lymphovascular Invasion, *E* Epithelial Component, *S* Sarcomatous Component, *ER* Estrogen Receptor, *PR* Progesterone Receptor, *CD3* Cluster of Differentiation 3, *CD4* Cluster of Differentiation 4, *CD8* Cluster of Differentiation 8, *FOXP3* Forkhead Box P3, *PD-1* Programmed Cell Death Protein 1, *PD-L1* Programmed Death-Ligand 1, *PD-L2* Programmed Death-Ligand 2

**Fig. 1:**
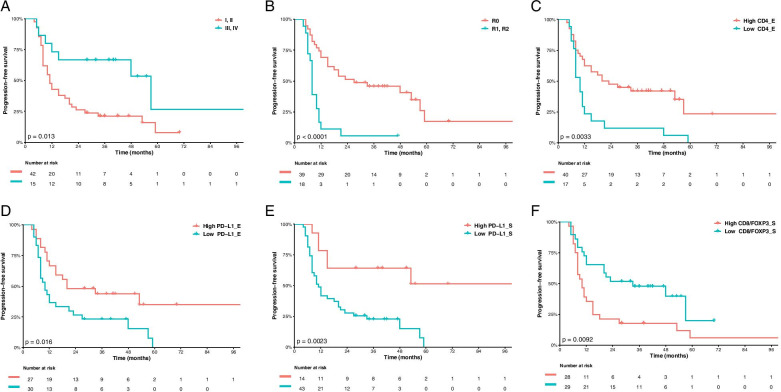
Progression-free survival (PFS) by: **A** stage; **B** residual disease status; **C** CD4_E status; **D** PD-L1_E status, **E** PD-L1_S status and **F** CD8/FOXP3_S ratio status. Residual disease after surgery was stratified into R0 (without residual disease) vs R1 (microscopic residual disease) and R2 (macroscopic residual disease). As for immunohistochemistry markers, Kaplan Meier curves for PFS were stratified by the median values as the cut-off for prognostic evaluation and divided into low vs high lymphocytic variable subsets. The blue solid line indicates patients with low values and the red solid line high values. Tick marks indicate censored data

**Fig. 2:**
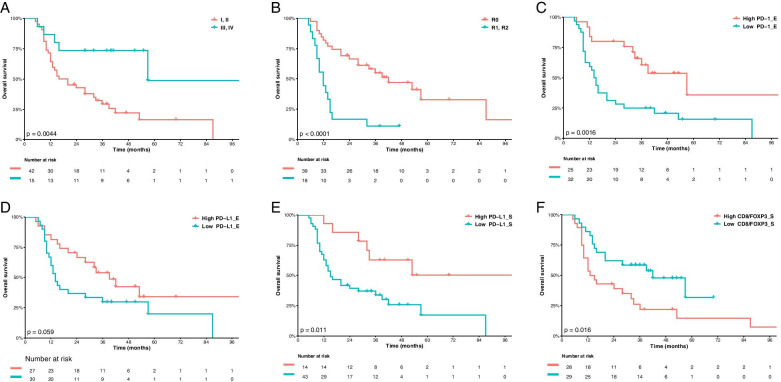
Overall survival (OS) by: **A** stage; **B** residual disease status; **C** PD1_E status; **D** PD-L1_E status, **E** PD-L1_S status and **F** CD8/FOXP3_S ratio status. Residual disease after surgery was stratified into R0 (without residual disease) vs R1 (microscopic residual disease) and R2 (macroscopic residual disease). As for immunohistochemistry markers, Kaplan Meier curves for OS were stratified by the median values as the cut-off for prognostic evaluation and divided into low vs high lymphocytic variable subsets. The blue solid line indicates patients with low values and the red solid line high values. Tick marks indicate censored data

Figures 1 and 2 show the Kaplan-Meier curves for PFS and OS according to the evaluated variables, respectively. Other complementary clinicopathological parameters did not influence the outcomes of PFS or OS (additional Tables 4 and 5).

## Discussion

Some of the main clinicopathological features of UCS in the current study are in line with previous reports of large cohorts performed by Matsuo et al. through multicenter studies and examining The Surveillance, Epidemiology, and End Results (SEER) program database [19–22]. In this regard, the mean age greater than 60 years and the LVI present in most patients are highlighted. Conversely, advanced-stage disease at diagnosis and heterologous sarcomatous component were more frequent characteristics in this cohort, perhaps due to the delay in diagnosis and local characteristics, respectively.

There is a growing body of evidence supporting the role of TME in the development and growth of solid tumors. Through pro-inflammatory cytokines actively secreted by tumor cells, leukocytes of the innate and adaptive immune system, including macrophages, neutrophils, NK cells, dendritic cells, mast cells, T and B lymphocytes, that infiltrate into the TME [23–26]. Thus, a better understanding of the composition of the lymphomononuclear infiltrate in TME has paved the way as biomarkers for a more personalized anticancer therapy. Many immunotherapeutic agents, including immunomodulators, vaccines, adoptive transfer of endogenous or genetically modified T cells, cytokines, and mainly immune checkpoint inhibitors (ICIs), have shown remarkably beneficial effects for better therapeutic response and increased survival in gynecologic cancers [27].

To the best of our knowledge, there is no previous published data assessing the predictive or prognostic role of TME markers in UCS so far. Likewise, studies on the characterization of TME in gynecological cancer are very scarce. Zhang et al. [28] investigated the prognostic impact of TME profile in 221 patients with endometrial cancer. Similar to our results, highly expressed CD4+ TILs were significantly associated with better OS and longer treatment-free interval and may be associated with chemosensitivity. Jong et al. [29] have recently reported 368 FIGO stage I–IV endometrial cancer patients with highly expressed CD8+ TILs, a marker for killer cytotoxic T cells, and a high CD8 +/FOXP3+ ratio was associated with better disease-free survival. FOXP3 is the most specific marker for Treg cells and, often associated with a negative impact on survival in several types of cancer, is likely to have an important role in suppressing anti-tumor immunity [30, 31]. Ore-Arce et al. [32] also reported that high CD8+ TILs was significantly associated with better 5-year OS in 68 women with FIGO stage I–IV endometrial cancer. Conversely, our current results suggested that high CD8/FOXP3_S and CD4/FOXP3_S ratios significantly yielded poorer survival outcomes.

Such conflicting findings might be strongly explained by tumor heterogeneity based on the histologic distribution of TILs at the tumor site. The aforementioned studies might have used TMAs that were built up with cores of diverse numbers, sizes and from distinct areas in the surgical samples (peri-tumoral or intra-tumor), consequently with different proportions of other immune cell subtypes that might have important roles in the TME (Myeloid-derived suppressor cells, MDSCs, Macrophages M2, granulocytes, B cells and so on). That said, these other subpopulations of TILs could influence the prognostic impact of the CD8+/FOXP3+ and CD4+/FOXP3+ ratios [33]. Salet and Elkordab [34] have suggested that Treg (FOXP3+) should be performed in subgroups based on their location in the tumor tissue and the current prognostic influence of each subgroup should be evaluated individually.

This cohort also suggested that highly expressed PD-L1, both in the epithelial and sarcomatous components, was found to be significant and independent marker for favorable PFS and OS. Likewise, highly expressed PD-1_E also showed a favorable association with OS in our cohort. PD-1/PD-L1 axis immune checkpoint signaling, known to play an important role in cancer progression and survival, is currently one of the most explored pathways in gynecological cancers [35]. Along with mismatch repair deficiency (dMMR), microsatellite instability (MSI) status and tumor mutational burden (TMB), PD-L1 has been identified as a potential predictive biomarker for endometrial cancer in some phase II clinical trials with immune checkpoint inhibitors [36, 37]. The cohort of 700 patients with uterine cancer performed by Engerud et al. [38] showed PD-L1 and PD-1 expression in 59 and 63% in primary tumors, respectively, with similar expression patterns across microsatellite stable (MSS) and MSI tumors. However, they did not influence survival outcomes.

Some other findings of this cohort suggest that the lymphocyte markers evaluated (CD3, CD4, CD8 and FOXP3), as well as PD-L1, PD-L2 and PD-1, seemed to be more highly expressed amidst the sarcomatous component. Therefore, UCS with sarcomatous dominance (defined as the proportion of the sarcoma component being greater than 50% in the primary tumor within all examined hysterectomy specimens), which was associated with shorter survival in previous reports [20, 21], may be targetable by immunotherapeutic agents. Unfortunately, the analysis of sarcomatous dominance is beyond the scope of the current study. Some data point to the fact that the more advanced the stage of solid tumors, the greater the expression of TILs markers in the TME favoring tumor progression [39].

The survival analyzes further exhibited that advanced stage (III/IV) and incomplete debulking are significantly associated with poorer PFS and OS outcomes. The negative prognostic impact of these clinicopathological variables has already been shown in other cohorts of carcinosarcoma [19, 20, 40, 41]. In a secondary analysis of a prior multicenter retrospective study, Matsuo et al. [42] suggested that LVI containing a sarcomatous component might be a predictor of decreased survival for women with UCS. However, LVI showed no significant association with survival in our cohort.

The strengths of this study lie in the novelty of the in-depth analysis of TME data in UCS by presenting the characteristics of the lymphomononuclear infiltrate correlating with clinicopathological features and evaluating the impact on survival. The study population is homogenous in that we only included patients with carcinosarcoma who underwent primary surgery and subsequently adjuvant chemotherapy with CP. Moreover, all surgical samples were double-checked by experienced pathologists. Lastly, a thorough descriptive presentation of clinicopathological variables was performed and multivariate analyzes reinforce the internal validity of the results.

The weaknesses of this study are strongly related to the fact that it is a retrospective analysis. So, some missing confounding factors may exist in the analysis. For example, even with well-established institutional protocols, the choice of adjuvant treatment with chemotherapy and or radiotherapy was at the discretion of the care providers. Additionally, the small sample may have been insufficient to ensure adequate power to detect differences in survival for some TME markers. Furthermore, molecular analysis was not performed in this study.

## Conclusion

This is possibly the first report to delve into the composition of TME in carcinosarcoma. Assessments of immune markers for progression and survival outcomes may have been impaired by the small sample. However, due to the increased prevalence of high expression of immune markers in this setting, the findings can respectfully provide some basis for formulating studies to evaluate novel therapeutic strategies with immunotherapeutic agents.

## Supplementary Information

**Additional file 1 **: **Table 1.** Baseline clinicopathological characteristics of eligible patients (*N* = 57). **Table 2**. Treatment data of the study population (*N* = 57). **Table 3.** Paired scores of immunohistochemistry markers for epithelial and sarcomatous components analyzed by the Wilcoxon signed-rank test. **Table 4.** Crude and adjusted Hazards Ratios for Carcinosarcoma progression-free survival (PFS) estimated by univariate analysis and multivariate analysis. **Table 5.** Crude and adjusted Hazards Ratios for Carcinosarcoma overall survival (OS) estimated by univariate analysis and multivariate analysis. **Figure 1**. Boxplots representing the distributions of the values of markers. **Figure 2**. Representative pictures of lymphocyte infiltration in uterine carcinosarcoma showing immunohistochemical staining of high CD3 +, CD4 +, CD8 +, FOXP3 +, PD-1 +, PD-L1 + and PD-L2 +. Original magnification: ×400 (×40 objective).

## Data Availability

The datasets generated during and/or analyzed during the current study are available from the corresponding author on reasonable request.
