# Supplementary material for: The prevalence and prognostic impact of tumor-infiltrating lymphocytes in uterine carcinosarcoma
Source: BMC Cancer. 2021 Dec 7;21:1306. doi: 10.1186/s12885-021-09026-6 (PMC8650400; doi:10.1186/s12885-021-09026-6)

Supplementary Material

**Additional table 1.** Baseline clinicopathological characteristics of eligible patients (N = 57)

| Clinicopathological features | N (%) |
| --- | --- |
| Age mean (SD)  < 60 years  ≥ 60 years | 65.3 (7.0)  17 (29.8%)  40 (70.2%) |
| BMI  ≤ 30 Kg/m²  > 30 Kg/m² | 31 (54.4%)  26 (45.6%) |
| Race  Caucasian  Non-Caucasian | 14 (25%)  42 (75%) |
| Stage  I/II  III/IV | 15 (26.3%)  42 (73.7%) |
| Histological subtype  Homologous  Heterologous | 12 (28.6%)  30 (71.4%) |
| LVI  Present  Absent | 25 (60.1%)  16 (39.9%) |
| HER2 overexpression  Present  Absent | 2 (3.5%)  55 (96.5%) |
| p53_E  Low  High | 19 (33.3%)  38 (66.7%) |
| p53_S  Low  High | 43 (75.4%)  14 (24.6%) |
| ER_E  Low  High | 44 (77.2%)  13 (22.8%) |
| ER_S  Low  High | 28 (50%)  28 (50%) |
| PR_E  Low  High | 41 (73.2%)  15 (26.8%) |
| PR_S  Low  High | 27 (48.2%)  29 (51.8%) |

BMI= Body Mass Index; LVI= Lymphovascular Invasion; E= Epithelial Component; S= Sarcomatous Component; ER= Estrogen Receptor; PR= Progesterone Receptor*.* Differences in absolute value correspond to missing data

**Additional table 2**. Treatment data of the study population (N = 57)

| Treatment | N (%) |
| --- | --- |
| Omentectomy  Yes  No | 27 (47.4%)  30 (52.6%) |
| Lymphadenectomy  Yes  No | 37 (65%)  20 (35%) |
| Adjuvant radiotherapy  Yes  No | 24 (42.8%)  32 (57.2%) |
| Residual disease  R1/2  R0 | 18 (31.6%)  39 (68.4%) |

**Additional table 3.** Paired scores of immunohistochemistry markers for epithelial and sarcomatous components analyzed by the Wilcoxon signed-rank test

| Immunohistochemistry factors | Epithelial component  Median IQR | | Sarcomatous component  Median IQR p-value | | |
| --- | --- | --- | --- | --- | --- |
| **CD3** | **1** | **5** | **10** | **39** | **<0.001** |
| **CD4** | **1** | **5** | **10** | **29** | **<0.001** |
| **CD8** | **1** | **5** | **10** | **25** | **<0.001** |
| **FOXP3** | **0** | **1** | **1** | **4** | **<0.001** |
| **PD1** | **0** | **1** | **1** | **1** | **0.002** |
| PDL1 | 1 | 10 | 5 | 19 | 0.080 |
| **PDL2** | **80** | **50** | **40** | **50** | **<0.001** |

IQR: Interquartile Range; CD3= *Cluster of Differentiation 3*; CD4= *Cluster of Differentiation 4*; CD8= *Cluster of Differentiation 8*; FOXP3= *Forkhead Box P3*; PD-1= *Programmed Cell Death Protein 1*; PD-L1= *Programmed Death-Ligand 1*; PD-L2= *Programmed Death-Ligand 2.* Significant P-values are emboldened

**Additional table 4.** Crude and adjusted Hazards Ratios for Carcinosarcoma progression-free survival (PFS) estimated by univariate analysis and multivariate analysis

| Clinicopathological features | Univariate analysis  HR 95%CI p-value | Multivariate analysis  HR 95%CI p-value |
| --- | --- | --- |
| BMI Kg/m² (> 30 vs ≤ 30) | 1.22 0.66-2.26 0.999 | 1.01 0.96-1.06 0.602 |
| Race (Caucasian vs non-Caucasian) | 0.86 0.40-1.83 0.695 | 1.10 0.52-2.30 0.799 |
| Omentectomy (Yes vs No) | 0.61 0.33-1.14 0.175 | 0.62 0.33-1.15 0.130 |
| p53_E (high vs low) | 1.06 0.55-2.06 0.852 | 0.81 0.41-1.61 0.552 |
| p53_S (high vs low) | 0.48 0.22-1.07 0.074 | 0.44 1.01-5.14 **0.048** |
| ER_E (high vs low) | 0.82 0.38-1.78 0.610 | 0.93 0.42-2.03 0.854 |
| ER_S (high vs low) | 1.18 0.64-2.17 0.594 | 1.17 0.64-2.14 0.618 |
| PR_E (high vs low) | 0.74 0.36-1.53 0.471 | 0.82 0.40-1.70 0.603 |
| PR_S (high vs low) | 1.31 0.71-2.41 0.388 | 1.27 0.69-2.34 0.436 |

BMI= Body Mass Index; LVI= Lymphovascular Invasion; E= Epithelial Component; S= Sarcomatous; ER= Estrogen Receptor; PR= Progesterone Receptor

**Additional table 5.** Crude and adjusted Hazards Ratios for Carcinosarcoma overall survival (OS) estimated by univariate analysis and multivariate analysis

| Clinicopathological features | Univariate analysis  HR 95%CI p-value | Multivariate analysis  HR 95%CI p-value |
| --- | --- | --- |
| BMI Kg/m² (> 30 vs ≤ 30) | 1.09 0.57-2.06 0.999 | 1.00 0.95-1.06 0.887 |
| Race (Caucasian vs non-Caucasian) | 0.86 0.40-1.83 0.695 | 1.03 0.47-2.26 0.930 |
| Omentectomy (Yes vs No) | 0.63 0.33-1.23 0.175 | 0.63 0.33-1.23 0.178 |
| p53_E (high vs low) | 1.02 0.51-2.02 0.852 | 0.81 0.40-1.63 0.553 |
| p53_S (high vs low) | 0.52 0.22-1.22 0.074 | 0.48 0.20-1.17 0.106 |
| ER_E (high vs low) | 0.67 0.28-1.61 0.610 | 0.78 0.32-1.90 0.590 |
| ER_S (high vs low) | 1.41 0.73-2.70 0.594 | 1.43 0.75-2.73 0.279 |
| PR_E (high vs low) | 0.64 0.29-1.41 0.471 | 0.73 0.33-1.61 0.442 |
| PR_S (high vs low) | 1.26 0.66-2.41 0.388 | 1.29 0.67-2.47 0.440 |

BMI= Body Mass Index; LVI= Lymphovascular Invasion; E= Epithelial Component; S= Sarcomatous; ER= Estrogen Receptor; PR= Progesterone Receptor

**Additional figure 1**. Boxplots representing the distributions of the values of markers


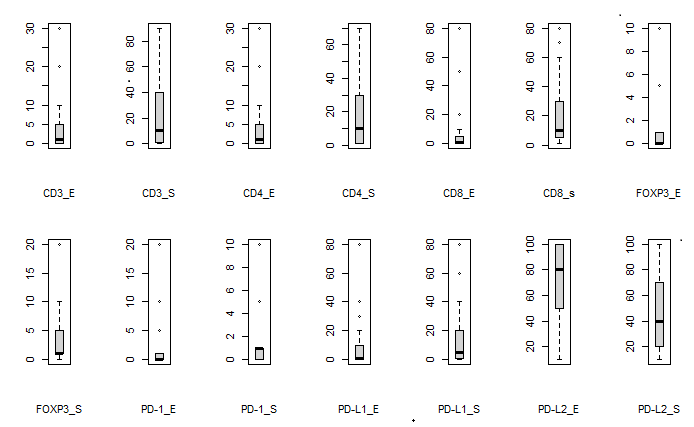


Additional figure 2. Representative pictures of lymphocyte infiltration in uterine carcinosarcoma showing immunohistochemical staining of high CD3 +, CD4 +, CD8 +, FOXP3 +, PD-1 +, PD-L1 + and PD-L2 +. Original magnification: ×400 (×40 objective).


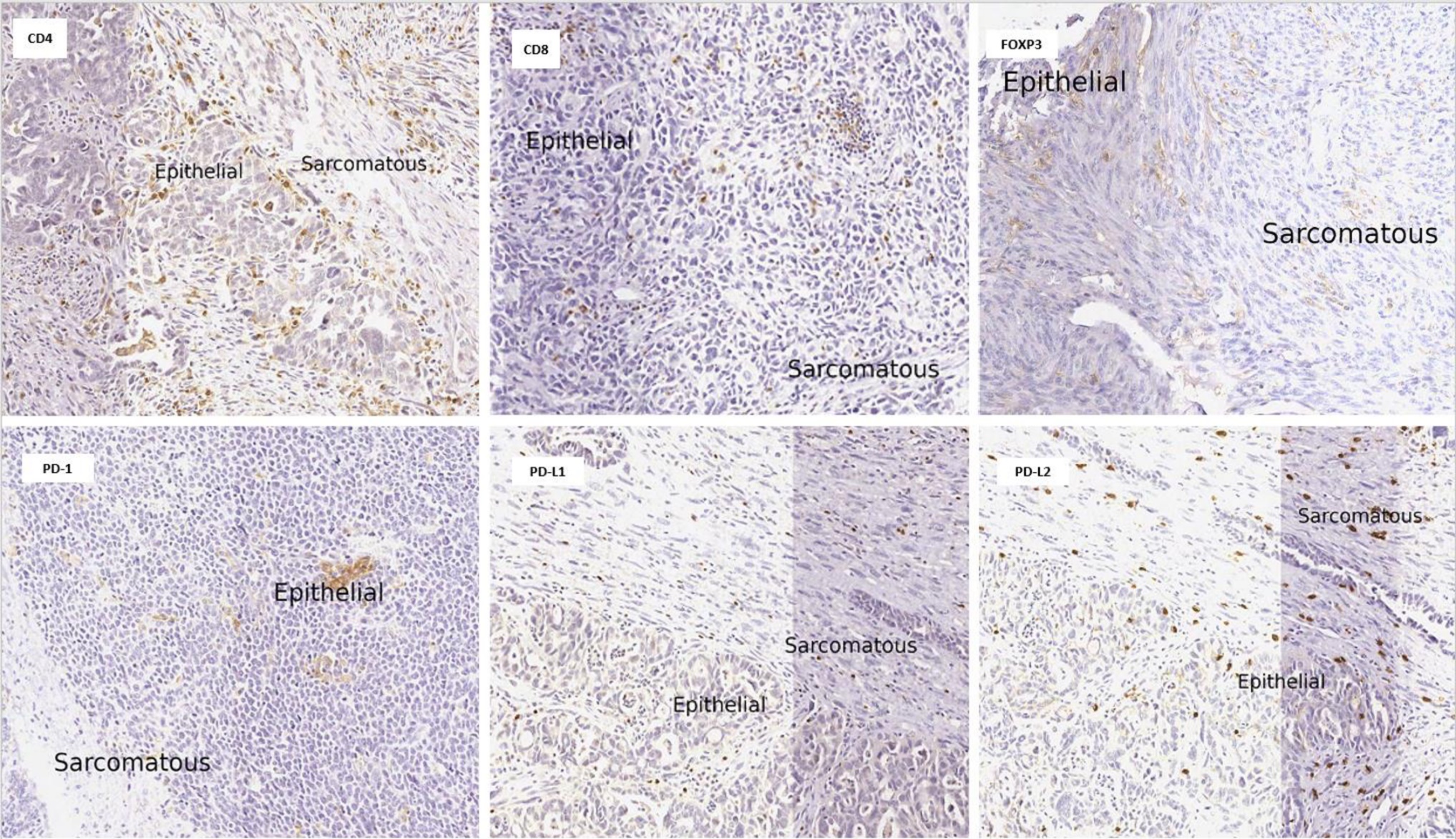

Supplement: Supplementary file 1 — Additional file 1 : Table 1. Baseline clinicopathological characteristics of eligible patients (N = 57). Table 2. Treatment data of the study population (N = 57). Table 3. Paired scores of immunohistochemistry markers for epithelial and sarcomatous components analyzed by the Wilcoxon signed-rank test. Table 4. Crude and adjusted Hazards Ratios for Carcinosarcoma progression-free survival (PFS) estimated by univariate analysis and multivariate analysis. Table 5. Crude and adjusted Hazards Ratios for Carcinosarcoma overall survival (OS) estimated by univariate analysis and multivariate analysis. Figure 1. Boxplots representing the distributions of the values of markers. Figure 2. Representative pictures of lymphocyte infiltration in uterine carcinosarcoma showing immunohistochemical staining of high CD3 +, CD4 +, CD8 +, FOXP3 +, PD-1 +, PD-L1 + and PD-L2 +. Original magnification: ×400 (×40 objective). [file 12885_2021_9026_MOESM1_ESM.docx]
